# In-situ directed energy deposition of Al based low density steel for automotive applications

**DOI:** 10.1038/s41598-023-49026-z

**Published:** 2023-12-11

**Authors:** Matěj Rott, Ying Li, Martina Koukolíková, Martina Šípová, Pavel Salvetr, Zbyšek Nový, Gerhard Wolf, Jan Džugan

**Affiliations:** 1 COMTES FHT a.s., Průmyslová 995, Dobřany, 334 41 Czech Republic; 2 Fraunhofer UMSICHT, An der Maxhütte 1, 92,237 Sulzbach-Rosenberg, Germany; 3 Department of Materials Science and Technology, West Bohemian University, Pilsen, Czech Republic

**Keywords:** Materials science, Structural materials

## Abstract

This work deals with the fabrication of one low density steel by mixing AISI S2 tool steel and AlSi10Mg powders using powder-based directed energy deposition (P-DED) technique. Two approaches of mixing powders were compared-continuous mixing during the process (in-situ) and mixing the powder prior to the process (premixed). The P-DED sample was characterised by a variety of techniques such as optical microscopy, scanning electron microscopy, electron backscatter diffraction, X-ray diffraction, and hardness measurement. Our findings demonstrate the successful achievement of steel with a 8 wt. % AlSi10Mg addition when two dissimilar powders are premixed, resulting in approximately 12% reduction in the density of S2 steel. Optimizing the powder feed rate and the ratio of AlSi10Mg powder contribute to an improvement of printability, eliminating materials separation, leading to a homogenous deposited part. Compared to the in-situ mixing case, the premixed process within the current process window generates a more homogeneous microstructure consisting of three phases: Ferrite, Fe_3_Al and Fe_3_AlC carbide. Whereas, the in-situ sample exhibits only two phases Ferrite and Fe_3_Al. The hardness of the premixed sample is found to be slightly higher compared to the in-situ sample.

## Introduction

The requirement to reduce the weight of components for automotive applications has become more stringent in recent years due to the need for improved fuel economy and reduced greenhouse gas emissions^1^. To meet this demand, developing lower density steels has emerged as a promising approach to directly decrease the weight of automotives. One widely indicated method to achieve low density steel is by adding a significant amount of Al to the steel^2^. However, conventional casting and rolling methods face several issues when producing low-density steels by adding Al-based alloys. These issues include elemental segregation at both micro and macro levels^3^, the formation of brittle and hard FeAl-type intermetallic^4,5^ and carbides^6,7^, and low efficiency combined with high costs for complex-structured components. More specifically, those brittle intermetallic and carbides will hamper the ductility of alloy at ambient temperature if without proper controlling distribution and morphology^8^. The emergence of additive manufacturing (AM) offers a promising alternative for manufacturing low-density steel with intricate structures that are difficult to be achieved conventionally. In addition, the AM process generates unique microstructural characteristics with finer grain structures and nonequilibrium phases due to the high cooling rate, which contribute to improved mechanical performance compared to conventionally manufactured counterparts^9,10^. Among the various AM techniques, directed energy deposition (DED) stands out with several unique advantages, particularly its ability to simultaneous use of multiple powdered materials, allowing for the creation of functionally graded structure, composites and customized materials combinations, i.e., low-density steel^11–13^.

AISI S2 tool steel is a water-hardening tool steel known for its extreme toughness and resistance to shock loading. The extreme toughness makes it well suited for applications where the components are subjected to dynamic and impact loads in the automotive and other transport technique sectors. The combination of AISI S2 tool steel with Al presents a promising solution for the development of robust, durable, and lightweight transport technique components, essential for improved automotive performance and efficiency. Potential applications encompass parts within transmission systems, high-performance brake components, or other wear-resistant components wherein both robustness and lightness are vital. However, there is a lack of available research on AM-S2 tool steel, particularly regarding the reduction of weight for S2 component fabricated by DED with the addition of Al element. In this study, two representatives powdered alloys AISI S2 and AlSi10Mg silumine were chosen to be mixed using DED technique in order to design a low-density steel for dynamically loaded conditions in automotive industries^6^. As mentioned previously, the DED machine allows for in-situ materials mixing by utilizing at least two continuous powder feeders. This enables the creation of specific compositions throughout the printing process and provides flexibility in adjusting the powder feed rate for each feedstock. In addition to in-situ mixing dissimilar powders, another approach is using premixed powder, which effectively combines multiple powders to obtain a high-quality printed part^14,15^. Therefore, two mixing approaches were compared in the present study, continuous in-situ mixing during the DED process (in-situ) and premixing the powders prior to the process (premixed). It is worth noting that while there is lack of research on AM-S2 tool steel, printed AM-AlSi10Mg has been extensively investigated^16–18^. The printability of AlSi10Mg has shown to be an issue^19^, as higher amount of Al leads to formation of brittle phase which have a negative effect on the mechanical properties. This suggests that a higher content of silumine in the steel might also cause challenges in terms of printability. Therefore, the addition of AlSi10Mg silumine needs to be carefully optimized during the DED process. Moreover, process parameters need to be fine-tuned to ensure proper mixing of multiple powders during fabrication and the formation of a homogeneous deposited part. Considering the uncertainties and limited data, establishing the process window and understanding its correlation with material behaviour are of great importance for the successful fabrication of low-density Al-based steel using the DED technique.

The objective of the present study is to investigate the feasibility of fabrication of a low-density steel by using powder-based directed energy deposition technique (P-DED). AISI S2 tool steel and AlSi10Mg powders were mixed in the process with compassion to the mixing the powder prior to the process. Two variables: content of addition of AlSi10Mg powder and powder feed rate impact on microstructure and quality of P-DED were investigated. Differences in printability, homogeneity in microstructure, phase continents and hardness are revealed and discussed here.

## Materials and methods

Two commercially gas-atomized powders from the manufacturer ©Sandvik were used in the current study-an AISI S2 steel with particle size distribution of 53–150 μm (mean particle size 109 ± 42 μm) and an AlSi10Mg silumine with particle size ranging from 63 to 150 μm (mean particle size 107 ± 36 μm). The particle size distribution and mean particle size for both powders were measured using Laser scattering particle size distribution analyser LA-960. The nominal chemical compositions of the used metal powder provided by the manufacturer are listed in Table 1. The parts were fabricated using a powder blown directed energy deposition system (InssTek MX-600, Daejeon, South Korea) equipped with 2 kW Ytterbium fibre laser source. The diameter of laser beam size was 1.6 mm. The Direct Metal Tooling (DMT) regime was used during fabrication of all parts. This regime changes the laser power in real-time based on the in-process camera response in order to control the height of the melt pool^20^. Therefore the layer height was set as a control parameter to 0.6 mm, as well as the laser scanning speed, which was set to 850 mm/min. Argon was used as both carrier and shielding gas during the deposition process. In order to investigate the feasibility of in-situ fabrication of low-density steel by mixing AISI S2 and AlSi10Mg, the printability process window needed to be established. Based on previous experience, three different weight ratios of powders were tested, 8%, 12% and 16% of AlSi10Mg. For each concentration, three different powder feed rate was varied, 3.25, 5.6 and 9.3 g/min. Carrier gas feed rate was set as a control parameter to 2.5 l/min. For in-situ process case, each powder, AISI S2 and AlSi10Mg was fed into the process from its separate supply container, namely that the mixing process was performed inside the powder delivery system. The laser power in the DMT mode was set in a range of 650–1000 W for all tested conditions. For comparison, premixed process was performed in which AISI S2 and AlSi10Mg powders were premixed using the Turbula 3D shaker mixer and then fed together into the fabrication process. In this case, the weight ratio of 8% AlSi10Mg was chosen based on the results of the in-situ process measurements. Various parts with dimensions of 15 × 15 × 15 mm^3^ were deposited on the C45 platforms with dimensions 100 mm × 100 mm with thickness of 20 mm. The actual chemical composition for various samples was measured using optical emission spectrometry. The presentative chemical composition of in-situ and premixed P-DED samples are listed in Table 1. More details for the process parameters are shown in Table 2.

**Table 1:** Nominal chemical composition of S2 and AlSi10Mg powders used and the actual chemical composition of as-deposited S2-AlSi10Mg specimen by P-DED.

Material	Fe	Si	Mn	Al	C	Mo	Mg
AISI S2 powder	Bal	1.1	0.6	0	0.48	0.6	–
AlSi10Mg powder	0.2	10.3	0.004	89	–	–	0.4
In-situ (I-2) P-DED	Bal	1.58	0.56	5.58	0.42	0.53	0.002
Premix (II-3) P-DED	Bal	1.67	0.53	6.61	0.38	0.51	0.002

**Table 2:** Defect incidence and density measured at in-situ and premixed process conditions.

Condition	Spec. no.	AlSi10Mg [*wt*.%]	Powder fed rate [g/min]	Defects				Area fraction [%]	Density [g/cm^3^]
				SC	LF	P	D		
In-situ	I-1	8	5.6			✓	✓	0.063	7.368
	I-2	8	3.3			✓		0.004	7.164
	I-3	8	9.3		✓	✓		0.166	7.385
	I-4	12	5.6			✓	✓	0.390	7.338
	I-5	12	3.3	✓		✓		0.044	7.003
	I-6	12	9.3		✓	✓	✓	0.316	7.335
	I-7	16	5.6	✓	✓	✓	✓	0.656	7.233
	I-8	16	3.3	✓		✓	✓	0.687	6.890
	I-9	16	9.3	✓	✓	✓	✓	0.264	7.409
Premixed	II-1	8	5.5			✓	✓	0.093	6.977
	II-2	8	4.4			✓	✓	0.142	6.905
	II-3*	8	5.5			✓	✓	0.034	7.057

*Carrier gas feed rate in sample II-3 was tuned from 2.5  to 1.25 l/min.

After fabrication, two batches of the as-deposited specimens for metallography analysis and hardness measurement were cut from the platform using an electro-discharge wire cutting machine. For metallography, the specimens were prepared by a standard procedure: grinding and subsequent polishing. The porosity and cracking were determined in the polished state using the NIKON EPIPHOT 200 optical microscope equipped with software for image analysis 3.22 NIS Element. To reveal the microstructure, the P-DED samples were etched in Nital 3% reagent for a few seconds. Metallographic observation and documentation of the microstructure were performed using the light microscope Carl Zeiss – Observer.Z1m and the scanning electron microscope JEOL IT 500 HR with an Octane Elite Super EDS analyser. X-ray diffraction (XRD) measurements were performed using BRUKER D8 DISCOVER diffractometer (Bruker AXS GmbH, Karlsruhe, Germany) with 1.5406 Å wavelength of Cu-Kα radiation at 40 kV and 40 mA. The increment was set to 0.025° in the range of 2θ from 35° to 105°. XRD data were collected four times at different regions with the polycapillary x-ray optics (2 mm in diameter). The phase composition and lattice parameters were evaluated by Rietveld refinement method^21^ using Topas software. The density was measured by the gravimetric method, according to Archimedes principle. The hardness profiles HV5 (load 5 kg) were measured using laboratory hardness tester Struers DuraScan 50 (EMCO-TEST Prüfmaschinen GmbH, Kuchl, Austria) equipped with ecos WorkflowTM software (EMCO-TEST Prüfmaschinen GmbH, Kuchl, Austria)^22^. The hardness was measured according to ISO 6507–1: Vickers hardness measurement. The measurement was performed from the platform along the building direction with the step size of 1 mm.

## Results and discussion

**Figure 1:**
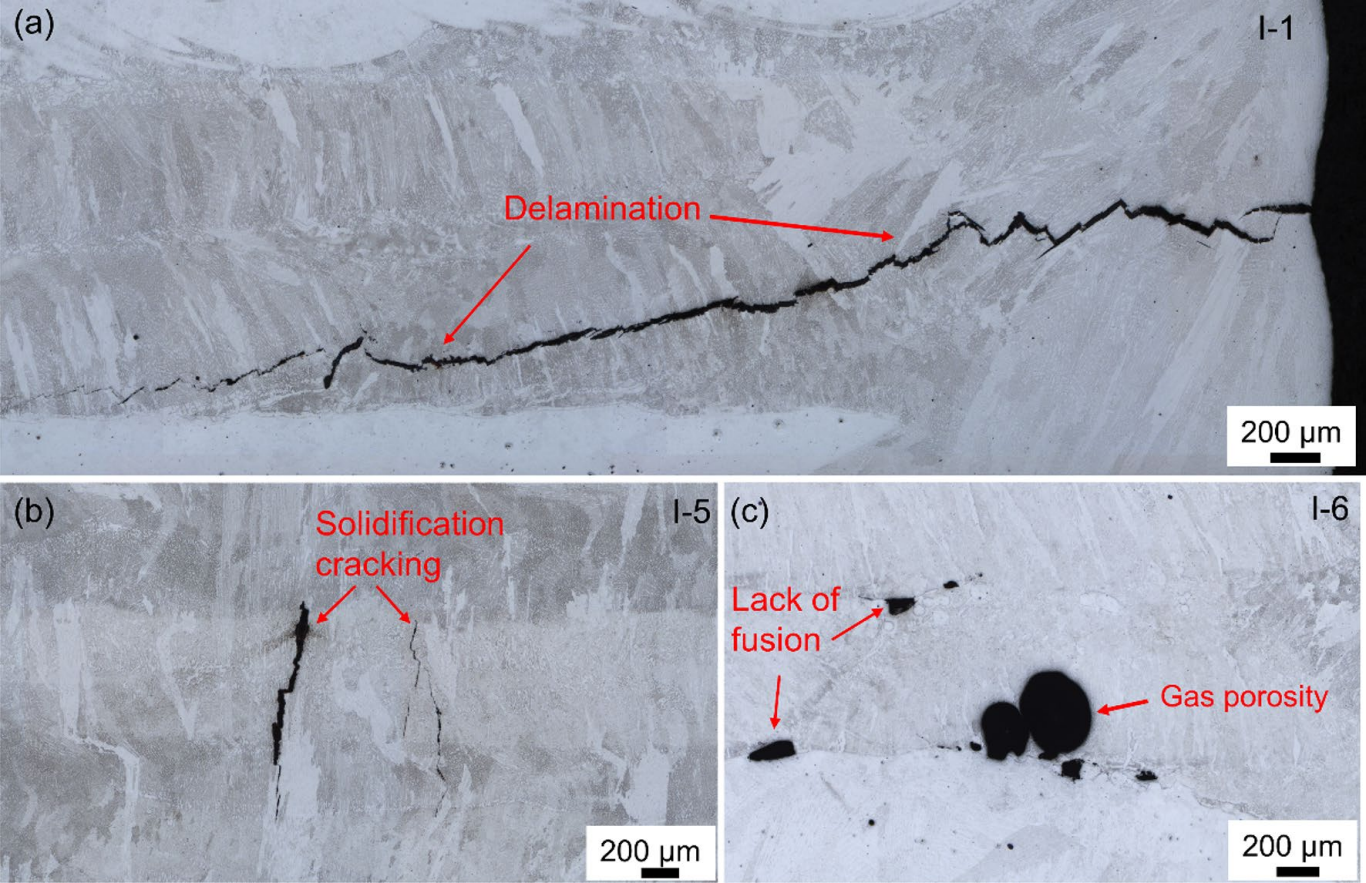
Comparison of defects produced by P-DED: (**a**) Delamination, (**b**) Solidification cracking and (**c**) Process-induced lack of fusion porosity and entrapped gas porosity.

Regarding the printability, in the first batch of in-situ fabrication, all specimens were successfully printed; however, majority of specimen exhibited cracking and/or delaminating from the substrate at various levels. Drawing from the available literature on additive manufacturing^23,24^, several types of defects have been observed, including solidification cracking (SC), gas-induced porosity (P), process-induced lack of fusion (LF), and delamination (D), as depicted in Fig. 1. Cracking and delamination are evident in regions dominated by AlSi10Mg, while adjacent areas enriched in steel demonstrate a resist to such defects. Summary of the occurrence of defects at various process parameters are clearly discernible in Table 2. In the current process window, the inclusion of AlSi10Mg powder is limited to 8 wt. %. Higher concentrations, such as 12% and 16%, inevitably lead to significant cracking and delamination despite optimized powder feed rates. Consequently, an 8 wt. % AlSi10Mg powder ratio was selected for premixing with AISI S2 tool steel powder for the premixed process condition. Compared to the in-situ process, specimens from the second batch, premixed with 8 wt. % AlSi10Mg powder, were successfully printed without obvious delamination from the substrate.

**Figure 2:**
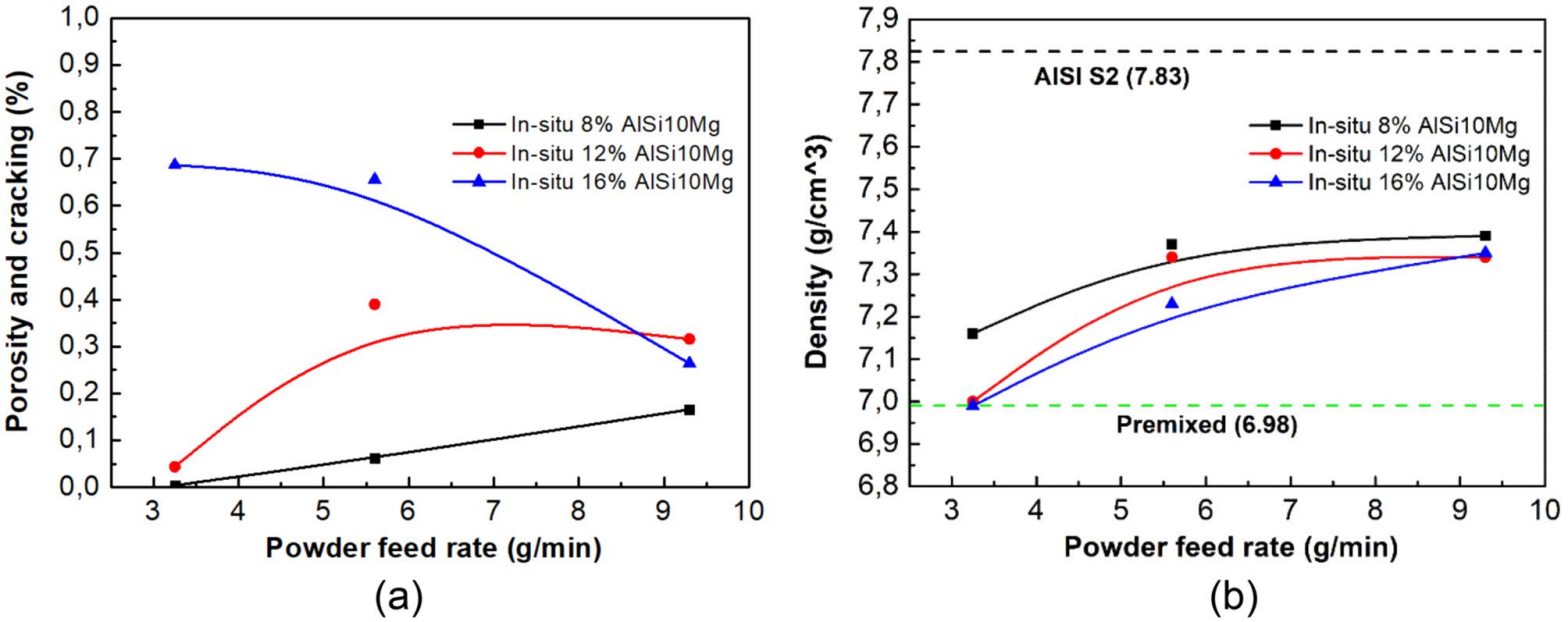
(**a**) Area fraction of defects and (**b**) density as a function of powder feed rate for AISI S2-AlSi10Mg mixed specimen at different additions of AlSi10Mg fabricated by P-DED.

To elucidate the interplay between defect content and process parameters, Fig. 2a plots the area fraction of defects against process parameters. It is evident that a higher AlSi10Mg ratio escalates defect incidence in in-situ materials, independent of powder feed rate. This restricted Al ratio in the mixture may be attributed to various factors including compound solubility, thermal expansion mismatch, and cooling rates. Concerning powder feed rate effect, its increment amplifies defect occurrence up to 12 wt. % AlSi10Mg, while beyond this ratio, a rise in powder feed rate mitigates defect appearance. This powder feed rate impact aligns with established findings^25,26^, where an inappropriate feed rate result in inconsistencies in the melt pool, potentially leading to increased porosity, lack of fusion between layers and other defects.

To clarify the impact of aluminium on reducing tool steel density, density of P-DED parts comprising both materials, with the relationships between density and powder feed rate at varying AlSi10Mg ratios is depicted in Fig. 2b. The data confirm that aluminium addition effectively diminishes the density of AISI S2 tool steel, with a more pronounced density reduction accompanying increased aluminium content. Within a fixed aluminium addition, powder feed rate also influences density, with lower rates favourably decreasing alloy density. Due to utilizing the gravimetric method for density measurement, the presence of porosity and cracks could potentially affect the results. Our examination of the correlation between defects and powder feed rate reveals that lower powder feed rates consistently enhance density reduction in in-situ samples at concentrations up to 12 wt. %. Beyond this, the apparent density reduction might stem from increased porosity and cracks. The in-situ I-2 sample outperforms other in-situ samples, displaying the least defects and density, with density reductions ranging from 5.9 to 12%. In contrast, premixed samples with an 8 wt. % AlSi10Mg addition mark an approximate 12% density decrease compared to S2 tool steel.

**Figure 3:**
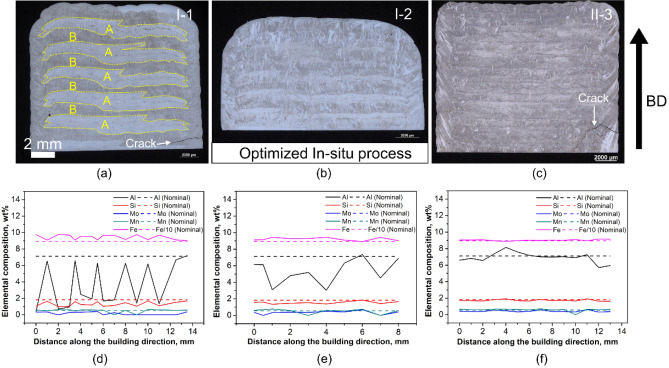
Optical micrographs of in-situ mixing (**a**) (**b**) and premixed samples (**c**) with the addition of 8% AlSi10Mg and its corresponding EDX line analysis showing the main elements distribution along the building direction.

Representative optical micrographs and the corresponding EDX analysis of samples from in-situ process and premixed process with the addition of 8% AlSi10Mg are shown in Fig. 3. For in-situ process, a higher powder feed rate gives rise to severe materials segregation, as shown in Fig. 3a. The specimens consist of separate layers of two regions: A–pure steel (highlighted by yellow dash lines) and B–mixture of S2 and AlSi10Mg silumine (marked in dark regions between steel regions), which was subsequently confirmed by the EDX analysis, as shown in Fig. 3d. The chemical composition of main alloy elements, especially Al and Fe, significantly deviate from the nominal value and fluctuate periodically due to the repetitive layer-wise characteristic. This deviation from the original composition ration is commonly observed in the fabrication of multi-materials with different densities, which is likely the result of the different particle flow dynamics presented under the same argon gas flow^15^. Decreasing the powder feed rate help reduce the materials separation, as shown in Fig. 3b. In-situ I-2 specimen with the lowest powder feed rate exhibits the highest microstructural and chemical homogeneity among all tested in-situ fabricated specimens. The corresponding EDX analysis confirm this improvement in chemical homogeneity, as presented in Fig. 3e.

Despite the optimization, the materials separation persists, leading to deviations from the desired composition. Other than further tuning laser power, optimizing powder particle size distribution may be an effective strategy to minimize materials separation and promote uniform composition. Considering the success of Li et al.,^15^ who rectified this issue by equalizing particle acceleration during the mixing of pure Cu and 4047 Al with disparate densities, we propose a focused examination into narrowing the particle size distribution. The adjustment of the size distribution, increasing for AlSi10Mg and decreasing for AISI S2 tool steel according to flow dynamic equations, holds promise. Under these refined conditions, the fabrication of samples with a higher aluminium content, maintaining compositional integrity, may indeed become achievable.

In addition, fine columnar grains with an average length of approximately 500 μm are observed to propagate through multiple deposited layers, which are typical grain microstructure produced by AM due to the high cooling rate^20,27^. Compared to in-situ process, more homogeneous microstructure is achieved for premixed condition, as shown in Fig. 3c. The corresponding EDX line analysis along the build direction shows all alloying elements nicely fit with the nominal value and fluctuate in a small range, which confirms the desirable fabrication combining of the two powders in this condition. The actual ratio of Al in the new deposited material is between 7–8 wt. %, reducing the density up to approximately 12% compared to the AISI S2 tool steel.

It should be noted that, the in-situ samples I-1 and I-2 revealed a sub-nominal aluminium (Al) content across the bulk, with nominal values only near the deposit's top. In contrast, the premixed samples displayed Al concentrations closely aligning with nominal values, exhibiting minimal deviation. This discrepancy suggests increased Al evaporation in in-situ samples, potentially contributing to their increased densities. During the in-situ mixing process, the disparate powders might not have amalgamated effectively, each interacting uniquely with the laser power. The present laser power setting, unsuitable for Al, might thereby exacerbate Al wastage. Conversely, in a premixed scenario, the unified powder blend likely interfaces more cohesively with the laser, mitigating evaporation and enhancing material conservation.

**Figure 4:**
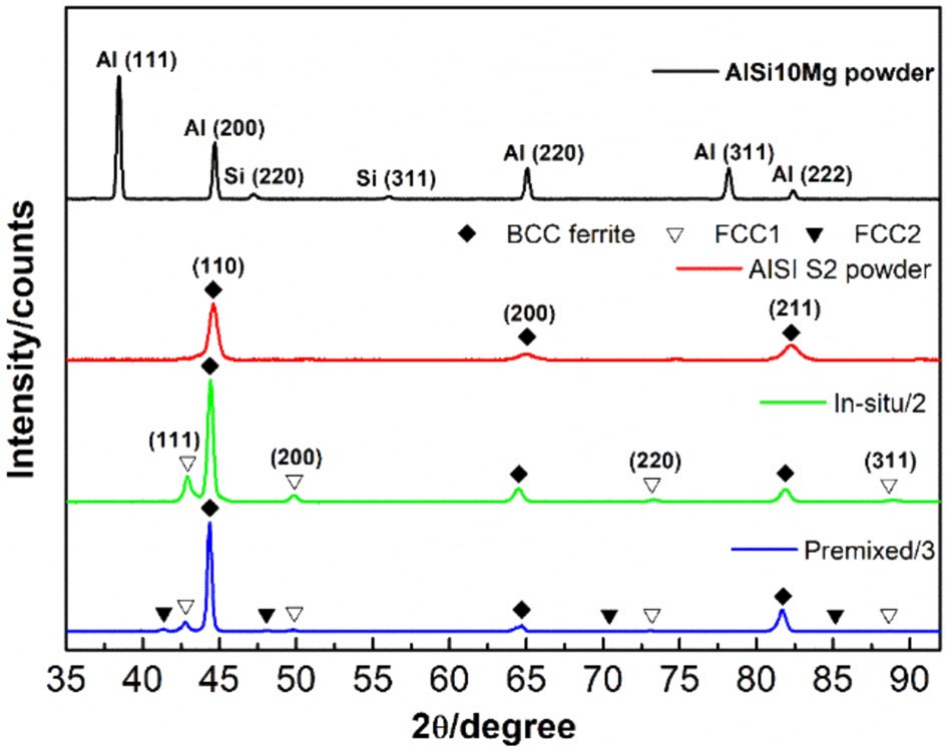
XRD patterns for in-situ and premixed P-DED specimens.

Figure 4 shows the presentative XRD patterns of AISI S2 and AlSi10Mg powder, in-situ and premixed P-DED produced parts. AISI S2 tool steel powder predominantly comprises of a body-centred cubic (BCC) ferrite phase in the matrix, with the strongest peak shown at 44.36°. After mixed with AlSi10Mg powder during the process, the new material consists of a new face-centred cubic (FCC 1) phase in addition to the BCC ferrite matrix. The volume fraction of FCC 1 phase is 27 ± 3%. As compared to the in-situ specimen, three phases are detected in premixed specimen. Another type of secondary FCC 2 phase with the typical diffraction peak observed at 42.8° is found along with the BCC ferrite matrix and FCC 1 phase. The FCC 1 phase accounts for 24 ± 6% while FCC 2 phase accounts for a lower amount of 7 ± 2%.

**Figure 5:**
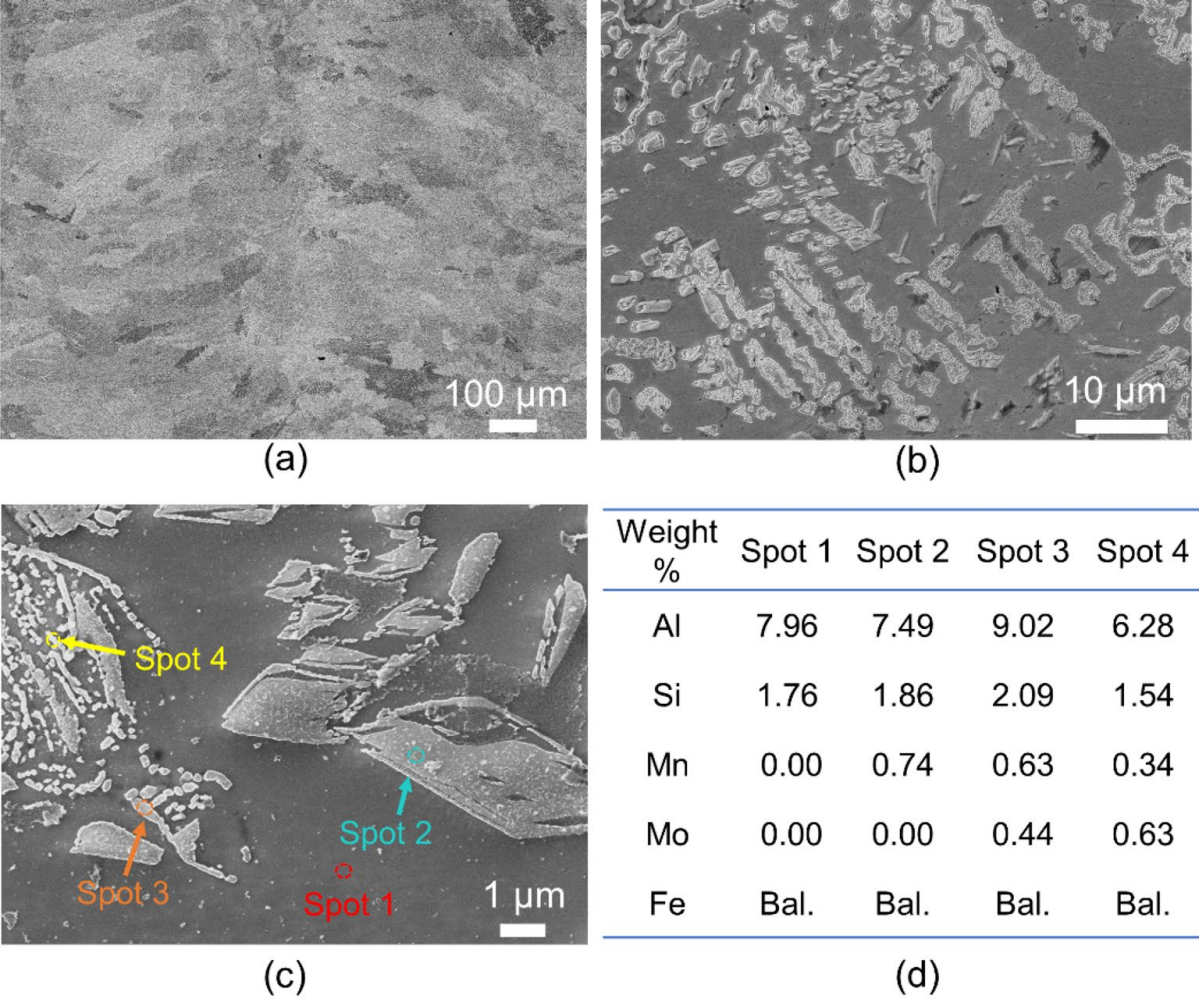
(**a**-**c**) SEM images of premixed II-3 specimen at different magnifications and (**d**) the chemical composition calculated from EDS results obtained from various phases marked in (**c**).

**Figure 6:**
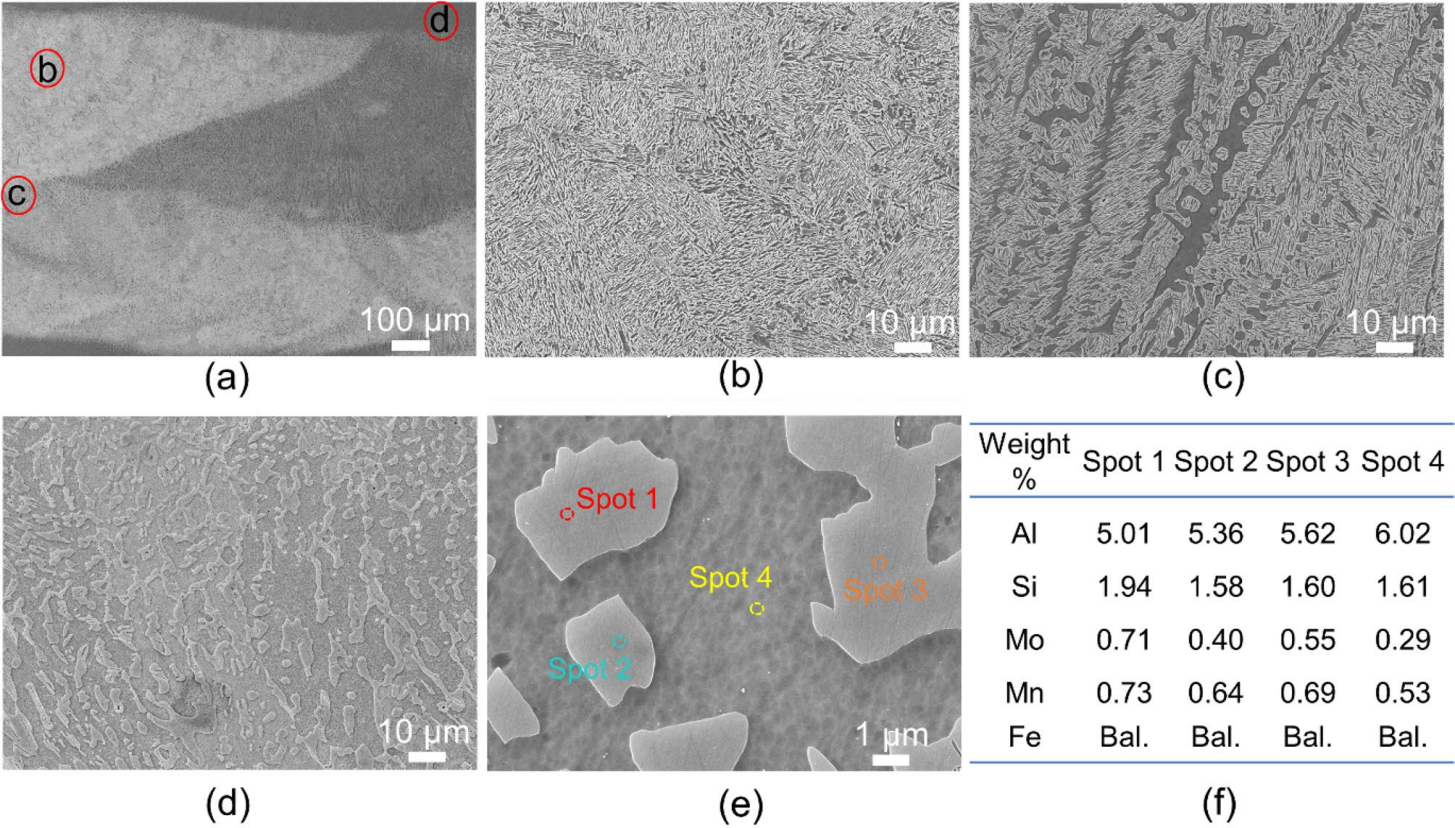
Microstructure of in-situ I-2 sample, (**a**) Overview showing material separation, (**b**-**d**) Zoomed-in regions specified in (**a**), (**e**) Identified phases within the in-situ I-2 sample, and (**f**) their chemical composition calculated from the EDS analysis.

Figure 5 and 6 shows microstructure of premixed II-3 and in-situ I-2 samples, respectively. As can be seen from Fig. 5a, homogenous microstructure is achieved in premixed sample, consistent with previous results. Complex phase constituents with various morphologies are observed at the higher magnifications, as shown in Fig. 5b-c. The chemical composition of all phases from EDS results does not show much difference, which makes it challenging to identify the specific FCC phases. Combined with XRD results and previous reports^28,29^, the elongated bands (spot 2) may correspond to Fe_3_Al, whereas, the finely dispersed nanometre-sized particles (near spot 3, 4) within the elongated bands is likely Fe_3_AlC. In comparison to the premixed sample, the in-situ variant presents a notably intricate microstructure, characterized by discernible material separation, as evident in Fig. 6a. A closer examination of areas highlighted in Fig. 6a reveals a microstructure contingent on location. For instance, region (b), where steel is predominant, displays similar microstructure to S2 steel, encompassing a blend of bainite, potential FeAl_3_ intermetallic, and traces of retained austenite. Conversely, region (c) recalls the prior observation with visible delta ferrite amidst the bainite-intermetallic blend, while region (d) illustrates an amplified presence of delta ferrite alongside the intermetallic amalgamation. The micrograph in (e) delves deeper, extracting a particular segment from (d), revealing intermetallic islands nestled amidst delta ferrite.

The observed variations in morphology, phase shape, and content among different samples are likely influenced by cooling rates and solidification conditions, given the array of process parameters employed in this study. Further investigations are essential to elucidate the relationships between specific process parameters, cooling rates, and solidification conditions. Notably, the microstructures presented here pertain to the as-deposited P-DED state. Given the rapid cooling rates inherent to P-DED, the method often yields metastable microstructures, complicating steel microstructure analysis. Post-processing heat treatments are imperative to refine and regulate the microstructure, particularly the Fe-Al intermetallic phase. By mastering its morphology and dispersion, this intermetallic phase can potentially be optimized for high-temperature applications, capitalizing on its robust high-temperature stability.

**Figure 7:**
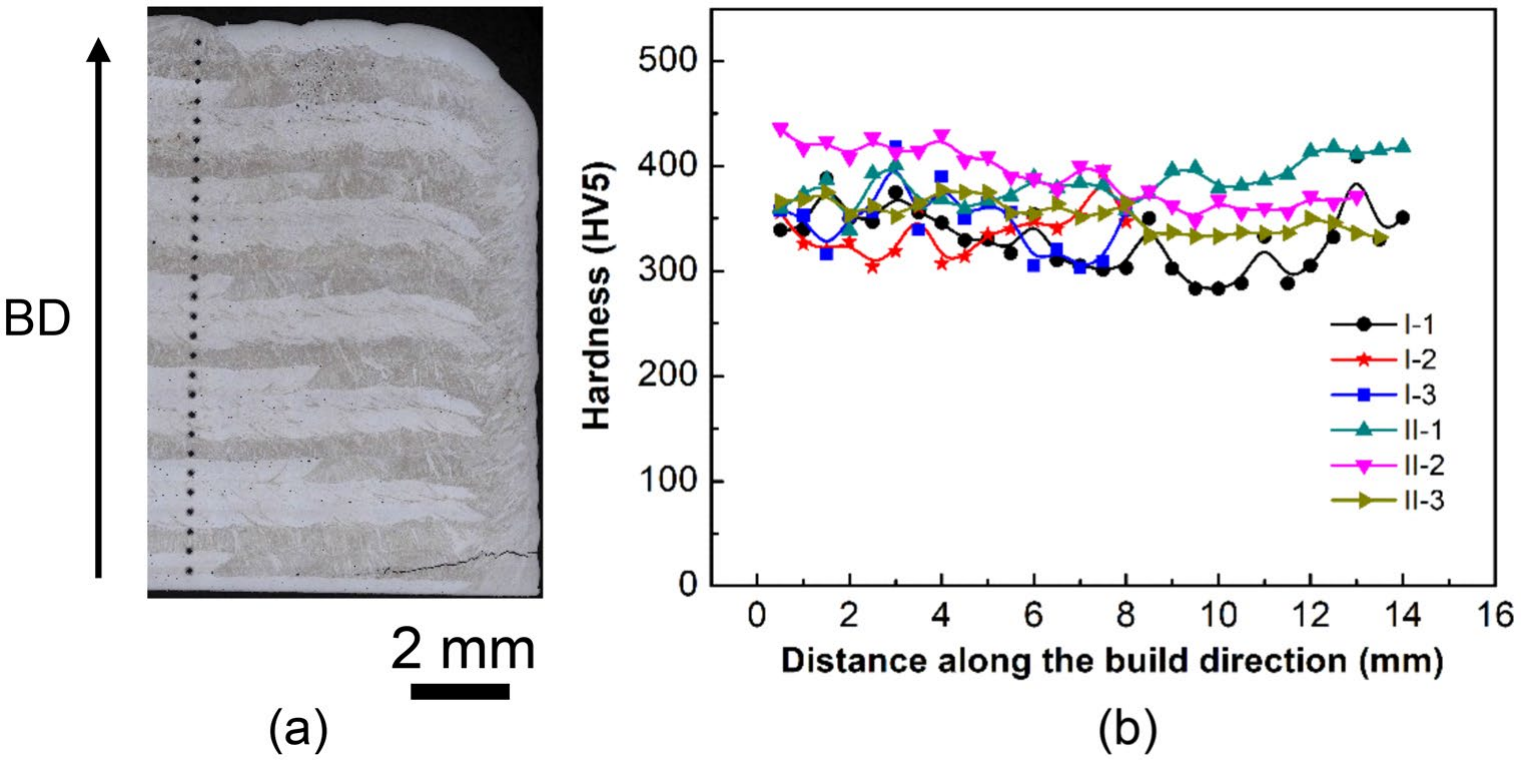
Hardness profile for in-situ and premixed specimen: (**a**) example testing approach of I-1 sample along the build direction (BD), (**b**) results of the HV5.

Figure 7 depicts the hardness profile as function of the distance from the bottom of the baseplate along the build direction. Summary of hardness results for all tested samples are listed in Table 3. It can be seen that, the values for in-situ tested sample are smaller than ones for premixed samples. A reduction in hardness in in-situ samples is linked to the scarce presence of Fe-Al intermetallic and carbide phases. Their inclusion generally enhance steel's strength and hardness, attributed to their high melting points and superior thermal stability^8,30^. In addition, a large scatter in the hardness results is observed for in-situ samples (I-1–3). The reduced and uneven hardness observed in the in-situ sample is likely attributed to the heterogeneous structure resulting from material separation. Each fluctuation in the structure mirrors a shift in composition. A more pronounced material separation results in more significant and abrupt changes in hardness. Within the large scatter, no clear and consistent dependency of the hardness on the location can be found for in-situ samples. Compared to the in-situ specimen, homogeneous values for hardness measurement can be obtained throughout the premixed deposited part due to the homogenous structure generated at this condition. Note that the hardness presented below was measured for as-deposited condition, and post-processing heat treatment is ongoing to tailor the microstructure and improve the hardness and other mechanical properties.

**Table 3:** Hardness of in-situ and premixed samples.

Sample	Mean Hardness (HV/5)	Standard Deviation
I-1	330	32
I-2	337	23
I-3	347	31
II-1	384	20
II-2	390	27
II-3	353	15

## Conclusions

In the present study, the printability, structural and chemical properties, as well as hardness of a new Al-based steel resulting from mixing AISI S2 tool steel and AlSi10Mg powders and processing them by P-DED technology were assessed. Two batches with different parameters were printed at in-situ alloying process and compared with premixed powder deposition process. The findings can be summarized as follows. P-DED samples with 8 wt.% of AlSi10Mg in the AISI S2 steel was successfully printed by premixing both powders, lowering the density by almost 12% compared to AISI S2 tool steel. Mixing higher amount of Al-based silumine alloy lead to cracking after printing process under current process window. In the case of in-situ P-DED samples, the reduction of density varied from 5.9 to 12% at various parameters and ratio of AlSi10Mg. In-situ powder mixing did not prove the capability of delivering a sufficiently homogenous mixture to the process with the present set-up, which resulted in a severe separation of two materials. Powder feed rate strongly affected the geometrical inaccuracy in the deposited part. However, the results differed for different parameter settings, suggesting a potential window for further optimization. On the contrary, premixed powder promoted a more homogenous structure, less cracking and better adhesion to the substrate. This approach of material preparation also lead to the lowest density of P-DED deposited materials – 6.98 g/cm^3^. The microstructure of premixed sample exhibited three phases: ferrite matrix (69 ± 7%), Fe_3_Al (24 ± 6%), and a low amount of Fe_3_AlC (7 ± 2%). Whereas, in-situ specimen consisted mainly ferrite matrix (73 ± 3%) and Fe_3_Al (27 ± 3%). No significant amount of Al containing carbides was observed in in-situ samples. These phase are commonly presented in the microstructure of Al alloyed steels after metallurgical preparation. Premixed sample exhibited a slightly higher and homogeneous value of hardness (375 ± 15 HV/5) compared to the in-situ sample (337 ± 23 HV/5). Our current study highlights the exceptional potential of multi-material directed energy deposition in the fabrication of low-density steel compared to the tradition metallurgical methods.

## Data Availability

The data that support the findings of this study are available from the corresponding authors upon reasonable request.

## References

[1] Liu M, Guo Y, Wang J, Yergin M (2018). Corrosion avoidance in lightweight materials for automotive applications. npj Mater. Degrad..

[2] Rana R, Liu C, Ray RK (2014). Evolution of microstructure and mechanical properties during thermomechanical processing of a low-density multiphase steel for automotive application. Acta Mater..

[3] Sunada H (1987). Mechanical properties of high carbon steels. Zair. Soc. Mater. Sci. Japan.

[4] Gschneidner K (2003). A family of ductile intermetallic compounds. Nat. Mater..

[5] Kuc D, Niewielski G, Bednarczyk I (2009). Structure and plasticity in hot deformed FeAl intermetallic phase base alloy. Mater. Charact..

[6] Kim H, Suh D-W, Kim NJ (2013). Fe–Al–Mn–C lightweight structural alloys: a review on the microstructures and mechanical properties. Sci. Technol. Adv. Mater..

[7] Sutou Y, Kamiya N, Umino R, Ohnuma L, Ishida K (2010). High-strength Fe-2OMn-Al-C-based alloys with low density. ISIJ Int..

[8] Kim SH, Kim H, Kim NJ (2015). Brittle intermetallic compound makes ultrastrong low-density steel with large ductility. Nature.

[9] Trosch T, Strößner J, Völkl R, Glatzel U (2016). Microstructure and mechanical properties of selective laser melted Inconel 718 compared to forging and casting. Mater. Lett..

[10] Deng D, Peng RL, Brodin H, Moverare J (2018). Microstructure and mechanical properties of Inconel 718 produced by selective laser melting: Sample orientation dependence and effects of post heat treatments. Mater. Sci. Eng. A.

[11] Demir AG, Previtali B (2017). Multi-material selective laser melting of Fe/Al-12Si components. Manuf. Lett..

[12] Rumman R, Lewis DA, Hascoet JY, Quinton JS (2019). Laser metal deposition and wire arc additive manufacturing of materials: An overview. Arch. Metall. Mater..

[13] Vaezi M, Chianrabutra S, Mellor B, Yang S (2013). Multiple material additive manufacturing - Part 1: a review: this review paper covers a decade of research on multiple material additive manufacturing technologies which can produce complex geometry parts with different materials. Virtual Phys. Prototyp..

[14] Banerjee R, Collins PC, Genç A, Fraser HL (2003). Direct laser deposition of in situ Ti–6Al–4V–TiB composites. Mater. Sci. Eng. A.

[15] Li W, Karnati S, Zhang Y, Liou F (2018). Investigating and eliminating powder separation in pre-mixed powder supply for laser metal deposition process. J. Mater. Process. Technol..

[16] Rosenthal I, Stern A, Frage N (2014). Microstructure and mechanical properties of AlSi10Mg parts produced by the laser beam additive manufacturing (AM) technology. Metallogr. Microstruct. Anal..

[17] Xiao Y (2022). Microstructure and mechanical properties of AlSi10Mg alloy manufactured by laser powder bed fusion under nitrogen and argon atmosphere. Acta Metall. Sin. English Lett..

[18] Gong J (2022). Microstructure and mechanical properties of AlSi10Mg alloy built by laser powder bed fusion/direct energy deposition hybrid laser additive manufacturing. Addit. Manuf..

[19] Gao Y (2019). Effect of processing parameters on solidification defects behavior of laser deposited AlSi10Mg alloy. Vacuum.

[20] Koukolíková M, Simson T, Rzepa S, Brázda M, Džugan J (2022). The influence of laser power on the interfaces of functionally graded materials fabricated by powder-based directed energy deposition. J. Mater. Sci..

[21] Rietveld HM (1969). A profile refinement method for nuclear and magnetic structures. J. Appl. Crystallogr..

[22] Kiran A (2022). Elevated temperature baseplate effect on microstructure, mechanical properties, and thermal stress evaluation by numerical simulation for austenite stainless steel 316L fabricated by directed energy deposition. Materials (Basel)..

[23] Sames WJ, List FA, Pannala S, Dehoff RR, Babu SS (2016). The metallurgy and processing science of metal additive manufacturing. Int. Mater. Rev..

[24] Galy C, Le Guen E, Lacoste E, Arvieu C (2018). Main defects observed in aluminum alloy parts produced by SLM: From causes to consequences. Addit. Manuf..

[25] Hicks C, Konkova T, Blackwell P (2020). Influence of laser power and powder feed rate on the microstructure evolution of laser metal deposited Ti-5553 on forged substrates. Mater. Charact..

[26] Kim TG, Shim DS (2021). Effect of laser power and powder feed rate on interfacial crack and mechanical/microstructural characterizations in repairing of 630 stainless steel using direct energy deposition. Mater. Sci. Eng. A.

[27] Wang YM (2018). Additively manufactured hierarchical stainless steels with high strength and ductility. Nat. Mater..

[28] Gschneidner K (2003). A family of ductile intermetallic compounds. Nat. Mater..

[29] Rana R, Liu C, Ray RK (2013). Low-density low-carbon Fe–Al ferritic steels. Scr. Mater..

[30] Stoloff NS, Liu CT, Deevi SC (2000). Emerging applications of intermetallics. Intermetallics.

